# MRD negativity: considerations for older adults with multiple myeloma

**DOI:** 10.1038/s41408-023-00939-y

**Published:** 2023-11-08

**Authors:** Hira Mian, Tanya M. Wildes, Christopher P. Venner, Rafael Fonseca

**Affiliations:** 1 Department of Oncology, McMaster University, Hamilton, ON Canada; 2 Department of Hematology/Oncology, University of Nebraska Medical Center/Nebraska Medicine, Omaha, NE USA; 3 Department of Medical Oncology, Centre for Lymphoid Cancers, University of British Columbia, Vancouver, BC Canada; 4 Division of Hematology and Medical Oncology, Mayo Clinic in Arizona, Phoenix, AZ USA

**Keywords:** Therapeutics, Prognosis, Health care

Minimal (or measurable) residual disease (MRD) negativity has emerged as one of the most important prognostic tools in multiple myeloma (MM) and is associated with significant improvements in progression-free survival (PFS) and overall survival [1]. Much of the focus on the role of MRD testing for prognosis and treatment selection has focused on younger, transplant eligible patients. However, MRD testing may be equally important among older transplant-ineligible patients with MM. We argue that MRD testing may prove to be a core component in the delivery of personalized and tailored treatment among older adults with MM. MRD testing may allow us to better balance both under-and over-treatment, a critical challenge in older adults with MM.

MRD negativity is associated with improved outcomes for older adults with MM [1]. Table 1 highlights key phase III clinical trials evaluating the impact of MRD negativity in this population (completed or ongoing). In the reported trials, MRD negativity was associated with improved PFS with further improved outcomes noted in patients with sustained MRD negativity [2]. Specifically in older adults, studies are planned that will incorporate MRD negativity as both an outcome and evaluate its potential for informing treatment modification or de-escalation.

**Table 1:** Key phase III trials in MM reporting on MRD negativity in transplant ineligible/deferred older adults.

Study	*N*	Treatment	MRD technique	Results
Published studies				
Pethema/GEM2010MAS65 [6] NDMM	163	1. VMP × 9 followed by Rd 2. Alt VMP/Rd × 18	Flow, 10^−5^	MRD negativity rates of 20% (arm 1) and 24% (arm 2) MRD-negativity was associated with a prolonged TTP (median not reached (NR) vs 35 months; *P* = 0.001) and OS (100% vs 72% at 3 years; *P* = 0.02) compared to MRD-positive patients
Myeloma XI trial [7] NDMM	297	1. CTDa 2. RCDa	Flow, 10^−4^	MRD negativity rates of 10.8% (arm 1) and 16.0% (arm 2) MRD negativity was associated with improved PFS (34 months vs 18 months; *P* < 0.0001, HR 0.44).
MAIA [2] NDMM	737	1. DRd 2. Rd	NGS, clonoSEQ assay, 10^−5^	MRD negativity: 28.8% (arm 1) and 9.2 % (arm 2) MRD-negative patients had improved PFS compared with MRD-positive patients (MAIA: HR, 0.15 [95% CI, 0.09–0.26]
ALCYONE [2] NDMM	706	1. Dara-VMP 2. VMP	NGS, clonoSEQ assay, 10^−5^	MRD negativity: 26.9% (arm 1) and 7.0% (arm 2) HR, 0.21 [95% CI, 0.15–0.30]; *P* < 0.0001
IKEMA [8] R/R	86 (age ≥70 years)	1. IsaKd 2. Kd	NGS, clonoSEQ assay, 10^−5^	MRD negativity: 23.1% (arm 1) and 11.8% (arm 2)
CLARION [9] NDMM	955	1. VMP 2. KMP	Flow, 10^−6^	MRD negativity: 15.5% (arm 1) and were 15.7% (arm 2)
GEM2005MAS65 [10] NDMM	260	1. VMP 2. VTD	Flow, 10^−4^	PFS: Arm 1 (MRD negative NR, MRD positive 28 months); Arm 2 (MRD negative 53 months, MRD positive 27 months); *P* = 0.01
ENDURANCE [11] NDMM	1087	1. VRd 2. KRd	Flow,	MRD negativity: 7.0% (arm 1) and were 10.0% (arm 2)
Trials in progress				
MagnetisMM- 6 (NCT05623020) NDMM	676	1. ElraRd 2. DRd	NGS, clonoSEQ assay, 10^−5^	Primary end point: MRD negativity at 12 months, PFS
MajesTEC 7 (NCT05552222) NDMM	1068	1. TecDRd 2. DRd	NGS, clonoSEQ assay, 10^−5^	Primary end points: PFS, MRD negativity at 12 months + CR
CEPHEUS (NCT04751877) NDMM	395	1. DRVd 2. RVd	NGS, clonoSEQ assay, 10^−5^	Primary end point: % of participants with negative MRD status
IMROZ (NCT03319667) NDMM	475	1. IsaRVd 2. RVd	NGS, clonoSEQ assay, 10^−5^	Primary end point: PFS Secondary end point: MRD negativity rate, sustained MRD negativity
CARTITUDE 5 (NCT04923893) NDMM	650	1. RVD-Ciltacel 2. RVD	NGS, clonoSEQ assay, 10^−5^	Primary end point: PFS Secondary endpoint: MRD negativity at 12 months + CR

*CR* complete response, *NDMM* newly diagnosed multiple myeloma patients, *NGS* next-generation sequencing, *NR* not reached, *PFS* progression-free survival, *R/R* relapsed/refractory, *TTP* time to progression.

Older adults with MM have traditionally had inferior outcomes compared to younger patients [3]. A significant challenge in treating older adults lies in accurately assessing a patient’s fitness status, which may not correlate with chronological age. A transplant ineligible older patient may range from a fit 74-year-old who is independent for all instrumental activities of daily living (IADLs) to a frail 81 year old with underlying co-morbidities and impairments in activities for daily living (ADLs). Furthermore, the treatment of older adults has been historically challenged by (1) limited number of available regimens achieving suboptimal response rates, and (2) by the increased risk of toxicity in this population. With newer therapeutic options that can induce deep responses, including MRD negativity, along with our ability to better identify those at greater risk for treatment-related toxicity, there is an opportunity to explore avenues for future personalization of treatment.

The ideal treatment for older adults should be highly effective, safe and with no enduring toxicity. Moreover, it should aim to minimize treatment burden, ideally with a finite duration, while improving patient quality of life. Even low-grade symptoms such as protracted fatigue from continuous immunomodulatory drugs can have a major impact on quality of life. The ideal treatment strategy should target not only specific disease characteristics but also patient specific characteristics, including frailty. A precise evaluation of disease response should allow for treatment titration including dose escalation, dose de-escalation or treatment discontinuation altogether.

MRD testing represents one of the most powerful prognostic tools in MM, offering the potential for a more precise approach to treatment. This approach applies across the entire spectrum of treatment options, including bispecific and CAR-T therapies. Moreover, insights from clinical trials suggest that treatments with a higher likelihood of achieving MRD negativity, along with agents adding only modest or minimal toxicity (e.g., monoclonal anti-CD38 antibodies [4]), should be pursued even in older adults. Combining this approach with improved risk stratification, particularly in identifying heterogeneity through frailty measures, we will be in a unique position to use MRD testing as a tool for personalizing care of older adults among both fit and frail older adults.

For instance, in an older adult receiving daratumumab–lenalidomide–dexamethasone therapy, achieving MRD negativity, especially sustained MRD negativity with a complete response, could prompt de-escalation of one of the agents or even therapy discontinuation, particularly if the current treatment is symptomatically burdensome. During this off-treatment phase, close monitoring would be essential, and if MRD resurgence occurs, therapy could be re-initiated, either with dara-len-dex or an alternative regimen. Proper tailoring using MRD testing should enhance quality of life, reduce treatment burden, and minimize the ongoing risk of treatment-related toxicity.

Despite its potential value, numerous key unanswered questions remain regarding the role of MRD testing in older adults with MM. We need further evidence to demonstrate that tailoring treatment based on MRD results improves patient outcomes, including quality of life among older adults. This evidence generation is necessary not only for fit older adults but also for the frail patient population. Among the frail group, low-intensity continuous treatments are often used, typically yielding low rates of MRD negativity. Thus, given the already high rates of toxicity among frail patients, thoughtful consideration and data are required to justify treatment escalation with potentially more potent agents to achieve higher rates of MRD negativity, while balancing the impact of treatment modification on the risk of treatment-related toxicity and quality of life.

Designing clinical trials involving older adults with MM to demonstrate the utility of MRD testing will pose unique challenges. It will be crucial to ensure that the eligibility criteria of these trials accurately reflect the real-world diversity of these patients, encompassing variations in comorbidities and functional status. Adequate sample sizes in these designed trials will also be essential to detect meaningful differences in MRD status and outcomes among older adults. Lastly, as MRD assessments often require long-term follow-up to assess their impact on patient outcomes, strategies to reduce attrition and maintain consistent follow-up over longer periods will be important. Ultimately, overcoming the challenges of designing trials in older adults, particularly to demonstrate the utility of MRD testing, demands a multidisciplinary approach involving geriatric specialists, oncologists, statisticians, and patient advocates. Striking a balance between scientific rigor and the unique needs and characteristics of real-world older adult patients is essential for obtaining clinically relevant results.

Additional challenges in MRD testing for older adults with MM include determining the optimal timing for MRD measurement. Unlike transplant-eligible patients with defined treatment blocks (induction, transplant, maintenance), many current treatment regimens for older adults are continuous, making it unclear when the ideal time for MRD assessment would be. This needs to be further analyzed with respect to the intended use of the MRD result. Discontinuation likely requires the establishment of durable MRD negativity while dose intensification may require more frequent testing at time points where an intervention would be re-planned. The optimal threshold (10^−5^ vs 10^−6^) also remains to be defined and may differ for fit older adults who may require dose escalation for MRD positivity versus among frail older adults where dose de-escalation is being considered for MRD negativity. Lastly, given that the risk of progression is dynamic, the role and feasibility of repeat bone marrow aspiration measurements for MRD testing in the context of dynamic frailty status [5] will also need to be considered. This consideration becomes particularly significant in older adults who may be more reluctant to undergo repeat invasive procedures such as bone marrow aspirations in the context of their overall health status, comorbidities, and personal preferences.

In summary, MRD testing stands as an exciting advancement in the field of MM, offering valuable insights into prognosis for older adults with MM. Additionally, while data remains somewhat limited, in specific scenarios such as discontinuing maintenance therapy, especially for patients encountering treatment-related toxicity, MRD-negative results could serve as a valuable tool in treatment decision-making. As emerging data continue to evolve, MRD testing holds the potential to help us fine-tune treatment strategies, optimizing both under- and over-treatment for the diverse population of older adults with MM.
